# Deprivation-Induced Plasticity in the Early Central Circuits of the Rodent Visual, Auditory, and Olfactory Systems

**DOI:** 10.1523/ENEURO.0435-23.2023

**Published:** 2024-02-09

**Authors:** Li Huang, Francesca Hardyman, Megan Edwards, Elisa Galliano

**Affiliations:** Department of Physiology, Development and Neuroscience, University of Cambridge, CB23EL Cambridge, United Kingdom

**Keywords:** audition, olfaction, plasticity, rodent, sensory deprivation, vision

## Abstract

Activity-dependent neuronal plasticity is crucial for animals to adapt to dynamic sensory environments. Traditionally, it has been investigated using deprivation approaches in animal models primarily in sensory cortices. Nevertheless, emerging evidence emphasizes its significance in sensory organs and in subcortical regions where cranial nerves relay information to the brain. Additionally, critical questions started to arise. Do different sensory modalities share common cellular mechanisms for deprivation-induced plasticity at these central entry points? Does the deprivation duration correlate with specific plasticity mechanisms? This study systematically reviews and meta-analyzes research papers that investigated visual, auditory, or olfactory deprivation in rodents of both sexes. It examines the consequences of sensory deprivation in homologous regions at the first central synapse following cranial nerve transmission (vision - lateral geniculate nucleus and superior colliculus; audition - ventral and dorsal cochlear nucleus; olfaction - olfactory bulb). The systematic search yielded 91 papers (39 vision, 22 audition, 30 olfaction), revealing substantial heterogeneity in publication trends, experimental methods, measures of plasticity, and reporting across the sensory modalities. Despite these differences, commonalities emerged when correlating plasticity mechanisms with the duration of sensory deprivation. Short-term deprivation (up to 1 d) reduced activity and increased disinhibition, medium-term deprivation (1 d to a week) involved glial changes and synaptic remodeling, and long-term deprivation (over a week) primarily led to structural alterations. These findings underscore the importance of standardizing methodologies and reporting practices. Additionally, they highlight the value of cross-modal synthesis for understanding how the nervous system, including peripheral, precortical, and cortical areas, respond to and compensate for sensory inputs loss.

## Significance Statement

This study addresses the critical issue of sensory loss and its impact on the brain’s adaptability, shedding light on how different sensory systems respond to loss of inputs from the environment. While past research has primarily explored early-life sensory deprivation, this study focuses on the effects of sensory loss in postweaning rodents. By systematically reviewing 91 research articles, the findings reveal distinct responses based on the duration of sensory deprivation. This research not only enhances our understanding of brain plasticity but also has broad implications for translational applications, particularly in cross-modal plasticity, offering valuable insights into neuroscientific research and potential clinical interventions.

## Introduction

Animals rely on sophisticated sensory organs to effectively perceive and interact with their surroundings. These sensory organs can convert various environmental stimuli, such as electromagnetic waves, mechanical pressure, and chemicals, into trains of action potentials that are relayed and computed in dedicated brain areas. The disruption of the sensory transduction cascade is a common occurrence attributable to factors such as trauma, ischemia, viral infection, and aging (Howarth and Shone, 2006; Worrall, 2007; Ryan et al., 2016; Boesveldt et al., 2017; Muth, 2017; Mullol et al., 2020). If left unattended, sudden sensory loss can significantly impact an individual’s behavior and well-being. Consequently, the nervous system must promptly adopt strategies to compensate for such losses. Unlike organs like the bones or skin, the adult brain cannot regenerate damaged peripheral sensors or central neurons, with only the olfactory system being a notable exception (Lepousez et al., 2013; Schwob et al., 2017). However, neurons can partially counteract the loss of sensory information by engaging a range of activity-dependent plasticity mechanisms (Carulli et al., 2011). These encompass both functional and structural changes at synapses, as well as adjustments in the intrinsic excitability and firing rates of neurons (Turrigiano, 2011; Wefelmeyer et al., 2016; Keck et al., 2017). Furthermore, glial cells also play a pivotal role in facilitating neuronal plasticity (Allen and Lyons, 2018; Sancho et al., 2021). The investigation of the mechanisms behind deprivation-induced adaptive plasticity across different timeframes, from immediate sensory loss to subsequent functional recovery, not only enhances our fundamental understanding of how neural circuits adapt to changing sensory inputs but also holds great significance for translational research in improving recovery after sudden sensory loss (Syka, 2002; Chen et al., 2011).

Since the seminal experiments of Hubel and Wiesel in monocularly deprived kittens (Hubel and Wiesel, 1963), multiple studies have dissected the mechanisms of deprivation-induced plasticity in animal models. This extensive body of work has largely examined the deprivation effects in primary sensory cortices during development and adulthood (Hensch, 2005; Feldman, 2009). While less emphasis has been placed on precortical regions (Jones, 2000; Duménieu et al., 2021), it is important to note that changes in these areas have a cascading impact on cortical adaptation and processing. Furthermore, existing studies in both the cortex and precortical areas have primarily focused on individual sensory modalities, making meaningful cross-modal comparisons challenging due to substantial experimental variability. This variability arises from factors such as the animals’ developmental stage, the experimental model, and heterogeneous methods for inducing adaptive plasticity through sensory deprivation (Maffei and Turrigiano, 2008; Pozo and Goda, 2010). Additionally, diversity in experimental design and result reporting complicates efforts to establish overarching principles governing the recruitment of different plasticity mechanisms in excitatory and inhibitory neurons, as well as glial cells. Synthesizing general principles is further hindered by anatomical and physiological diversity in the various sensory pathways, including differences in sensory organ complexity, transduction mechanisms, and the number of precortical relays.

**Figure 1.**
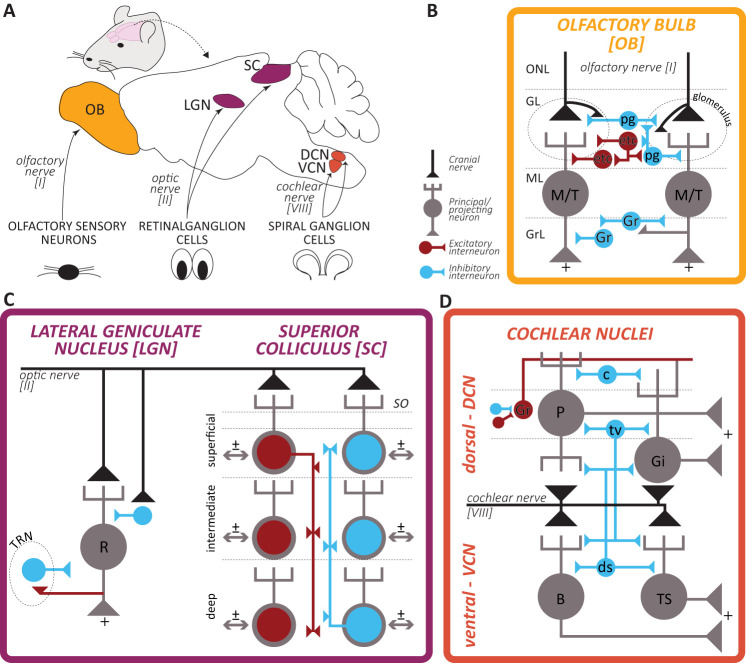
Architecture of the early olfactory, visual, and auditory pathways. ***A***, Schematic representation of the mouse brain and location of the olfactory bulb (OB), lateral geniculate nucleus (LGN; dorsal, dLGN; and ventral, vLGN, combined), superior colliculus (SC), dorsal and ventral cochlear nuclei (DCN and VCN), and their respective cranial nerves input from the sensory organs. ***B–D***, Simplified circuitry of the early central circuits processing olfactory, visual, and auditory information (summarized and adapted from Oertel and Young, 2004; Shepherd, 2004; Campagnola and Manis, 2014; Kosaka and Kosaka, 2016; Weyand, 2016; Duménieu et al., 2021; Liu et al., 2022). Black line and triangle indicate the cranial nerve ending, gray cells are principal neurons projecting outside these early circuits to higher processing areas, red and blue cells are local interneurons, respectively, excitatory and inhibitory. For ease of representation, the many central inputs to these circuits are not depicted. In the bulbar circuit: ONL, olfactory nerve layer; GL, glomerular layer; ML, mitral layer; GrC, granule cells layer; M/T, mitral/tufted cell; pg, periglomerular cell; etc, external tufted cell; gr, granule cell. In the geniculate circuit: R, relay neuron; TRN, thalamic reticular nucleus. In the collicular circuit: SO, stratum opticum; note that the exact circuitry has not been fully resolved, and that all layers send projections outside the SC. In the cochlear nuclei circuit: P, pyramidal (or fusiform); Gi, giant cell; B, bushy cell; TS, T-stellate cell; ds, d-stellate cell; tv, tubercoloventral (or vertical) cell; c, cartwheel cell; Gr, granule cell with its axon called parallel fiber.

To overcome these complications, this study focuses on deprivation-induced plasticity in anatomically homologous subcortical hubs in the olfactory, visual, and auditory pathways in rodents. The olfactory bulb (OB), lateral geniculate nucleus and superior colliculus (LGN and SC), and the dorsal and ventral cochlear nuclei (DCN and VCN) receive the primary synapse in the brain made by the respective cranial nerves (Fig. 1). While their circuit architectures differs granularly, these five circuits share multiple features (Oertel and Young, 2004; Shepherd, 2004; Campagnola and Manis, 2014; Kosaka and Kosaka, 2016; Weyand, 2016; Duménieu et al., 2021; Liu et al., 2022). Principal neurons receive glutamatergic inputs from the olfactory, optic, or cochlear nerves and send their axons to higher processing areas (from which they receive feedback projections which are beyond the scope of this study). Importantly, all five circuits heavily feature local inhibitory interneurons modulating information transfer, with additional excitatory interneurons described in both OB and DCN.

While we acknowledge the extensive literature on sensory deprivation in diverse animal models, especially primates and carnivores (King and Parsons, 1999; Kupers and Ptito, 2014), our systematic search was tailored to rodents. This choice allowed us to maintain an ethologically focused approach while still ensuring the inclusivity necessary to capture a significant number of articles across all sensory modalities. To disentangle early developmental plasticity from activity-dependent plasticity in self-sufficient animals, we exclusively considered in vivo deprivation studies in postweaning rodents (i.e., juveniles and adults).

Our systematic search across two databases returned 91 articles which employed visual, auditory, or olfactory deprivations spanning durations from 30 min to over a year and utilized a range of experimental techniques from transcriptomics to behavioral assays. This meta-research study pursued two primary objectives. First, we aimed to elucidate the characteristics of the literature and provide recommendations for designing, executing, and reporting sensory deprivation experimental approaches in rodent models. Second, we sought to identify commonalities in precortical plasticity across the three senses, facilitating the synthesis of generalizable principles. Such insights can inform analogous approaches in other systems (e.g., in cortex, following sensory enrichment), as well as translational research on recovery from sudden sensory loss.

## Materials and Methods

### Inclusion criteria

We included papers which met all of the following criteria: (a) rodent animal models (mouse, rat, guinea pig, hamsters, gerbils) of either sex, (b) primary research, (c) English language, (d) sensory deprivation performed (d′) in vivo (d″) in animals which had been weaned for the entire duration of the deprivation, and (e) plasticity investigated at the location of first central synapse after cranial nerve (i.e., olfactory bulb, lateral geniculate nucleus, superior colliculus, dorsal and ventral cochlear nuclei, trigeminal nucleus). The choice of a weaning as a cutoff was driven by the absence of information about full-adulthood milestones (e.g., puberty and sexual maturity) and the predominant use of juvenile animals in ex vivo electrophysiological studies.

### Search strategy

Two separate searches, without any time constraints on publication date, were performed on 22 November 2021 in PubMed and Scopus using the Boolean search strings detailed below:

“*((mouse) OR (rat) OR (gerbil) OR (guinea pig) OR (rodent))*

*NOT (cross-modal)”*

***AND*** *((sensory deprivation) OR (auditory deprivation) OR (auditory deafferentation) OR (visual deprivation) OR (dark exposure) OR (enucleation) OR (retinal lesions) OR (olfactory deprivation) OR (odor deprivation) OR (naris occlusion) OR (nostril occlusion) OR ((trimming) AND (whiskers)) OR ((plucking) **AND** (whiskers)) OR ((pruning) AND whiskers)) OR (ear plug) OR ((cauterised) AND (naris)) OR ((cauterised) **AND** (nose)) OR ((cauterised) AND (olfactory)) OR (nose plug) OR ((naris) AND (closure)) OR ((whisker) AND (deprivation)))*

***AND*** *((superior colliculus) OR (lateral geniculate nucleus) OR (visual thalamus) OR (cochlear nucleus) OR ((trigeminal nucleus) AND (whisker)) OR (olfactory bulb))*

***AND*** *((plasticity) OR (adaptation) OR (adaptive) OR (experience-dependent) OR (homeostatic) OR (synaptic scaling) OR (compensatory) OR (activity-dependent plasticity) OR (firing-rate homeostasis) OR (intrinsic excitability))*

For Scopus, the string was modified by adding “TITLE-ABS-KEY [“ at the start, and brace {} instead of brackets ()].

### Study selection

**Figure 2.**
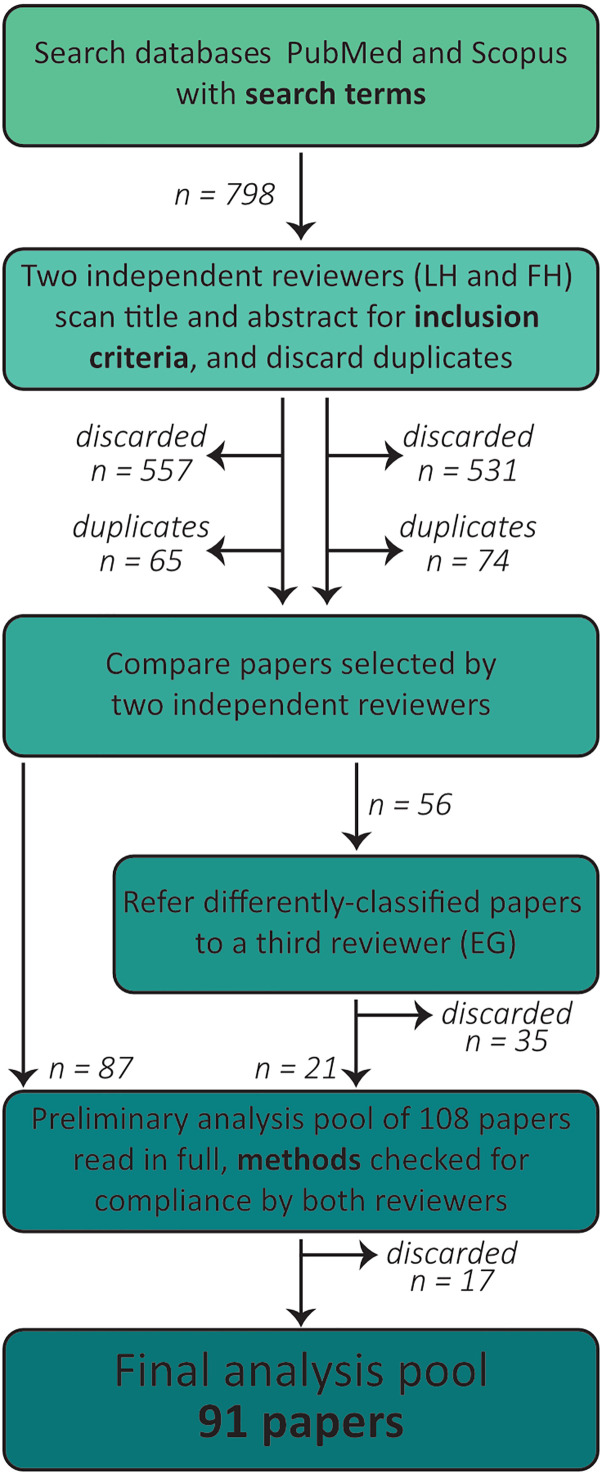
Strategy for literature search and papers selection. Flowchart indicating the number of articles returned by the parallel searches in the databases PubMed and Scopus using the search terms detailed in the text and their subsequent selection by two independent scrutineers.

In the first screening stage, the title and abstract of the research papers from both databases were scrutinized by two independent reviewers (L.H. and F.H.) to ensure compliance with the inclusion criteria a–c. Duplicates (research papers found in both databases) were removed. Disagreements were resolved by a third reviewer (E.G.). In the second screening stage, the full text of shortlisted studies was considered against the inclusion criteria d and e (see Inclusion criteria and Fig. 2).

### Data extraction and availability

Prior to the search, a list of relevant information to be extracted from each selected paper was agreed upon. These included the following: (1) publication year, (2) rodent species, (3) sex, (4) age at deprivation onset, (5) deprivation type and its possible reversibility, (6) method of deprivation, (7) duration of deprivation, (8) experimental method used to probe plasticity, (9) cell type in which the plasticity was investigated, and (10) main findings. Papers meeting inclusion criteria were then carefully screened for this information, which was collated into a master spreadsheet. The dataset is available under a CC BY copyright at https://doi.org/10.1523/ENEURO.0435-23.2023.

### Statistical analysis

Statistical analysis was performed in Prism (GraphPad) and R. Data were checked for normality, one-way ANOVA with Tukey’s post hoc correction for multiple comparisons was used to assess differences in deprivation duration, and chi-squared tests were used to compare proportions. Significance was set to *p* < 0.05. For the meta-analysis, mean and SD as well as *n* number were extracted manually from the relevant papers and submitted to meta-analysis in R using the “metafor” and “meta” packages.

## Results

### Summary of included papers

Two search strategies were employed to screen articles (Fig. 2). PubMed yielded 579 articles, while Scopus returned 219. The inclusion of tactile sensing (whisking) was initially considered but later discarded due to the limited number of relevant papers (only five). After screening the abstracts of the 798 retrieved research papers to remove duplicates, both independent reviewers identified 87 articles that met the inclusion criteria. The 56 papers selected by only one reviewer underwent a third-party review, resulting in the exclusion of 35 papers. The remaining 108 papers underwent a full-text review, and 17 of them did not meet the inclusion criteria. Ultimately, 91 papers were included in the analysis.

### Literature characteristics: publication trends over time

**Figure 3.**
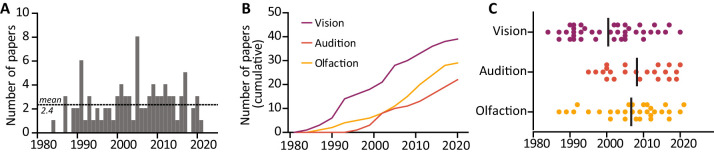
Publication trends over time. ***A***, No significant trend in the volume of published primary research articles over the 1984–2021 time period. Dotted line represents mean number of publications per year (2.4 mean, 1.7 standard deviation). ***B***, Cumulative distribution of papers published over the 1984–2021 period for each investigated sensory modality. ***C***, Distribution of papers over time for each investigated sensory modality. Dots are individual studies, line indicates median year. Purple, vision; orange, audition; yellow, olfaction.

First, we analyzed the publication trends over time across vision, audition, and olfaction in the qualifying papers which investigated deprivation-induced plasticity in cranial nerve receiving areas. We found no significant trend in the number of publications over time (Fig. 3*A*, simple linear regression; *R*^2^ = 0.06; *F*_(1,36)_ = 2.34; *p* = 0.14). The mean publications per year was 2.4 papers (SD = 1.7). The number of studies investigating the visual system was higher than in olfaction and audition (Fig. 3*B*, cumulative distributions). The median publication year was calculated for each sense, and vision studies appear to be older than olfaction and audition (Fig. 3*C*, vision, *n* = 39, median, 2002; audition, *n* = 22, median, 2009; olfaction, *n* = 30, median, 2009). In summary, we found that the field of deprivation-induced plasticity is steadily productive, with studies focusing on vision being more numerous.

### Features of the experimental models

**Figure 4.**
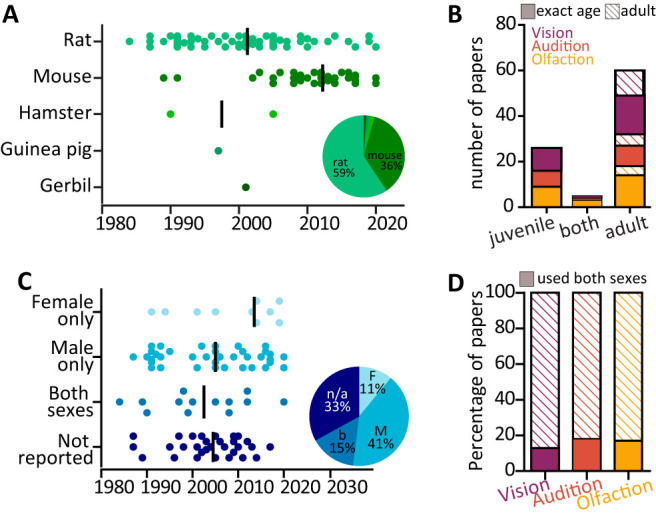
Animal models. ***A***, Distribution of rodent species used over time (1984–2021). Dots are individual studies; line indicates median publication year. Inset: pie chart reporting the percentages of the 91 selected studies using rats (59%), mice (36%), and other rodents (5% split among hamsters, guinea pigs, and gerbils). ***B***, Number of papers using only juvenile animals (postweaning to P40; 26 papers), both juvenile and adult animals (5 papers), and only adult animals older than 40 postnatal days (filled rectangles) or stated “adult” without specifying the age (striped rectangles) across vision (purple), audition (orange), and olfaction (yellow). ***C***, Distribution of rodent sex used over time (1984–2021). Dots are individual studies; line indicates median publication year. Inset: pie chart reporting the percentages of the 91 selected studies using females only (11%), males only (41%), both sexes (15%), or failing to report the sex of the used animals (33%). ***D***, Proportion of papers using both sexes (filled rectangles) across the three sensory modalities: purple, vision, 13%; orange, audition, 18%; yellow, olfaction, 17%. Striped rectangles include studies which used only one sex or failed to report the sex used.

Next, we analyzed the use of animal models over years and across senses. Most studies used either rats (*n* = 54, of which 35% Wistar and 30% Sprague Dawley) or mice (*n* = 33, of which 48% C57Bl6 wild-type, 15% wild-type animals in other genetic background, and 30% genetically modified mice). In line with general trends in the field and the advent of numerous commercially available genetically modified lines, the use of mice has significantly increased since the early 2000s (mouse median publication year, 2012; rat median publication year, 2001). Only three studies used other rodent models, namely, gerbils (*n* = 1), guinea pigs (*n* = 1), and hamsters (*n* = 2; Fig. 4*A*). In terms of age, most studies reported using fully adult animals (i.e., animals reported as older than 40 d or “adult”; 65/91 papers). Some studies used exclusively juvenile rodents aged between weaning and postnatal day 40 (26 papers in total, 10 vision, 7 audition, 9 olfaction). These potentially fall within critical windows of heightened plasticity (Hensch, 2005; Andrade-Talavera et al., 2023), but they are a minority and equally distributed across the three senses (Fig. 4*B*). Regarding the rodents’ sex, studies most commonly used exclusively male rodents (41%), and unfortunately many articles did not report the animals’ sex (33%). However, recent years saw an increase of studies using female rodents (female only, *n* = 10, median publication year, 2014; male only, *n* = 37, median publication year, 2005; both sexes, *n* = 14, median publication year, 2003; not reported, *n* = 30, median publication year, 2005; Fig. 4*C*). Finally, we found no significant differences in the proportion of studies using both sexes among the three sensory modalities, with similar proportions of papers using both sexes (vision, 13%; audition, 18%; olfaction, 17%; chi-squared test; *χ*^2^(2) = 1.04; *p* = 0.59; Fig. 4*D*).

### Features of the experimental paradigm: diverse methods to induce sensory deprivation

**Figure 5.**
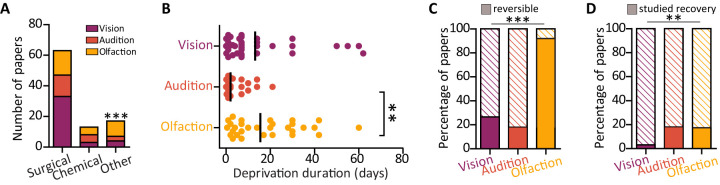
Deprivation method. ***A***, Number of papers using surgical, chemical, or other (plugs, patches) deprivation methods. ***B***, Minimum deprivation duration in days used in each study (individual dots) and mean duration (black line). ***C***, Proportion of papers using reversible deprivation methods (filled rectangles) across the three sensory modalities: purple, vision, 26%; orange, audition, 18%; yellow, olfaction, 90%. ***D***, Proportion of papers which used a reversible method to induce deprivation and investigated recovery (filled rectangles) across the three sensory modalities: purple, vision, 3%; orange, audition, 18%; yellow, olfaction, 17%. **p < 0.01, ***p < 0.001.

Next, we focused on how visual, auditory, and olfactory deprivations were induced. The most common method to induce sensory deprivation involves lesioning the peripheral sensory organ via surgical or chemical approaches (surgical lesion, *n* = 63; chemical lesion, *n* = 13; lesion methods combined, 82%; Fig. 5*A*). Other less invasive methods were sense-specific and included using nose and ear plugs for, respectively, olfactory and auditory deprivation and dark rearing for visual deprivation (other methods, *n* = 17, 18%; Fig. 5*A*). The minimum duration of deprivation significantly differed among sensory modalities (one-way ANOVA; *F*_(2,87)_ = 5.13; *p* = 0.0078), with the audition field adopting significantly shorter deprivation durations (4.9 d ± 5.7 d) compared with olfaction (18.05 d ± 15.65 d) and a trend of shorter duration compared with vision (13.16 d ± 2.791 d; Tukey’s post hoc audition vs olfaction, *p* < 0.01; audition vs vision, *p* = 0.09; vision vs olfaction, *p* = 0.36; Fig. 5*B*).

We also assessed how many studies used a reversible deprivation method which allows investigating the circuit recovery while leaving it anatomically intact. Such methods include nose and ear plugs and eye patches or dark rearing. We found that, compared with vision and audition, a larger fraction of olfactory papers used reversible methods, chiefly the insertion and removal of a nose plug (vision, 26%; audition, 18%; olfaction, 90%: chi-squared test; *χ*^2^(2) = 126; *p* < 0.0001; Fig. 5*C*). Consequently, olfactory and auditory studies often investigated the functional recovery after cessation of the sensory deprivation (vision, 3%; audition, 18%; olfaction, 17%: chi-squared test *χ*^2^(2) = 12.72; *p* = 0.0017; Fig. 5*D*). It is noteworthy that while not every olfactory study using nose plugs investigated functional recovery, every auditory paper employing reversible deprivation techniques, that is, ear plugs, studied the circuit recovery after plug removal.

### Features of experimental paradigm: deprivation-induced plasticity readouts

**Figure 6.**
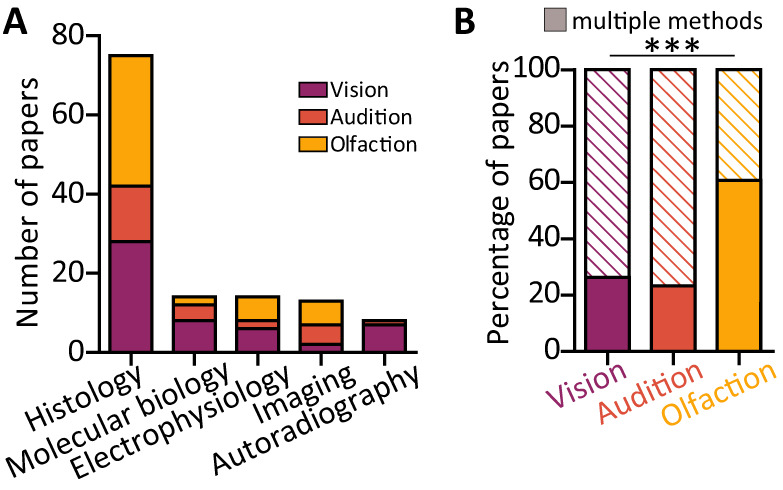
Plasticity readouts. ***A***, Number of papers across the three sensory modalities using each investigative methodology. ***B***, Proportion of papers using more than one method to investigate the effects of deprivation (filled rectangles) across the three sensory modalities: purple, vision, 26%; orange, audition, 23%; yellow, olfaction, 60%. ***p < 0.001.

After confirming that sensory deprivation is induced in two broadly similar ways across the three sensory modalities—permanent lesions or reversible removal of the sensory stimuli—we proceeded to assess how the consequences of such sensory deprivations were investigated. Across all sensory modalities, the predominantly employed experimental technique was histology (*n* = 75), followed by molecular biology assays (*n* = 14), electrophysiological recordings (*n* = 14), and functional imaging in live tissue (*n* = 13). Although histology was the most commonly used technique, the proportion of studies employing each technique is different across the senses (chi-squared test; *χ*^2^(8) = 38.34; *p* < 0.0001). Notably, autoradiography is used almost exclusively by vision researchers (except for 1 auditory study; *n* = 7; Fig. 6*A*). We observed more pronounced differences when we analyzed the number of studies employing more than one method to probe deprivation-induced plasticity in the target circuits. While approximately a quarter of papers focusing on visual and auditory early brain areas used more than one technique, over half of olfactory papers investigated the consequences of smell deprivation in the olfactory bulb using multiple methods (vision, 26%; audition, 23%; olfaction, 60%: chi-squared test; *χ*^2^(2) = 36.51; *p* < 0.0001; Fig. 6*B*).

### Features of experimental paradigm: definition of cell types

**Figure 7.**
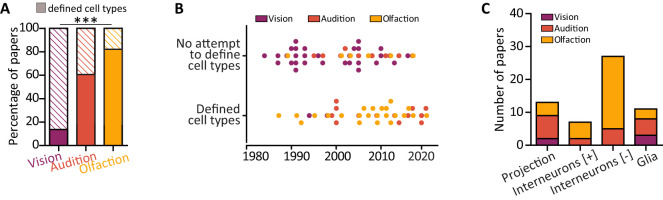
Definition of cell types. ***A***, Proportion of papers defining the cell types where the deprivation-induced plasticity was investigated (filled rectangles). ***p < 0.001. ***B***, Distribution of papers over time split by the lack/presence of cell type definition. Dots are individual studies; purple, vision; orange, audition; yellow, olfaction. ***C***, Among the studies which defined cell types, number of papers investigating deprivation-induced plasticity in projection/principal neurons, excitatory (+) or inhibitory (−) interneurons, and glia across the three sensory modalities. Note the lack of vision papers focusing on interneurons.

As experience-dependent plasticity is cell type specific (Pozo and Goda, 2010; Fox and Stryker, 2017), we next assessed on which cell types the 91 selected studies focused their investigation on. We broadly grouped cell types into glia and neurons and further subdivided the neuronal class into principal neurons, whose axon projects out of the circuits where their soma resides (i.e., relay neurons in the lateral geniculate nucleus and superior colliculus, bushy and T-stellate cells in the ventral cochlear nucleus, fusiform and giant cells in dorsal cochlear nucleus, mitral/tufted cells in the olfactory bulb), and excitatory or inhibitory interneurons, whose processes are fully contained in the local circuit. We found that a significant number of studies across all three sensory modalities, but more pronouncedly in vision, did not define the cell types that they investigated, either because they looked at area-wide measures or because they did not classify the types/subtypes of cells that they assessed (number of studies defining cell types: vision, 13%; audition, 59%; olfaction, 80%: chi-squared test; *χ*^2^(2) = 93.96; *p* < 0.0001; Fig. 7*A*). Given the historical popularity of visual deprivation (Fig. 3*C*; mean publication year, 2002), we analyzed the trend over time for studies to define cell type (Fig. 7*B*). As the median year for cell type definition is 2009, we confirmed that defining cell types has become more routine in recent years (e.g., the two most recent papers in vision both defined cell types; Jaepel et al., 2017; Huh et al., 2020), but not fully penetrant since long-established practices in each field seem to somewhat linger.[Fig eN-NWR-0435-23F8]

**Figure 8. eN-NWR-0435-23F8:**
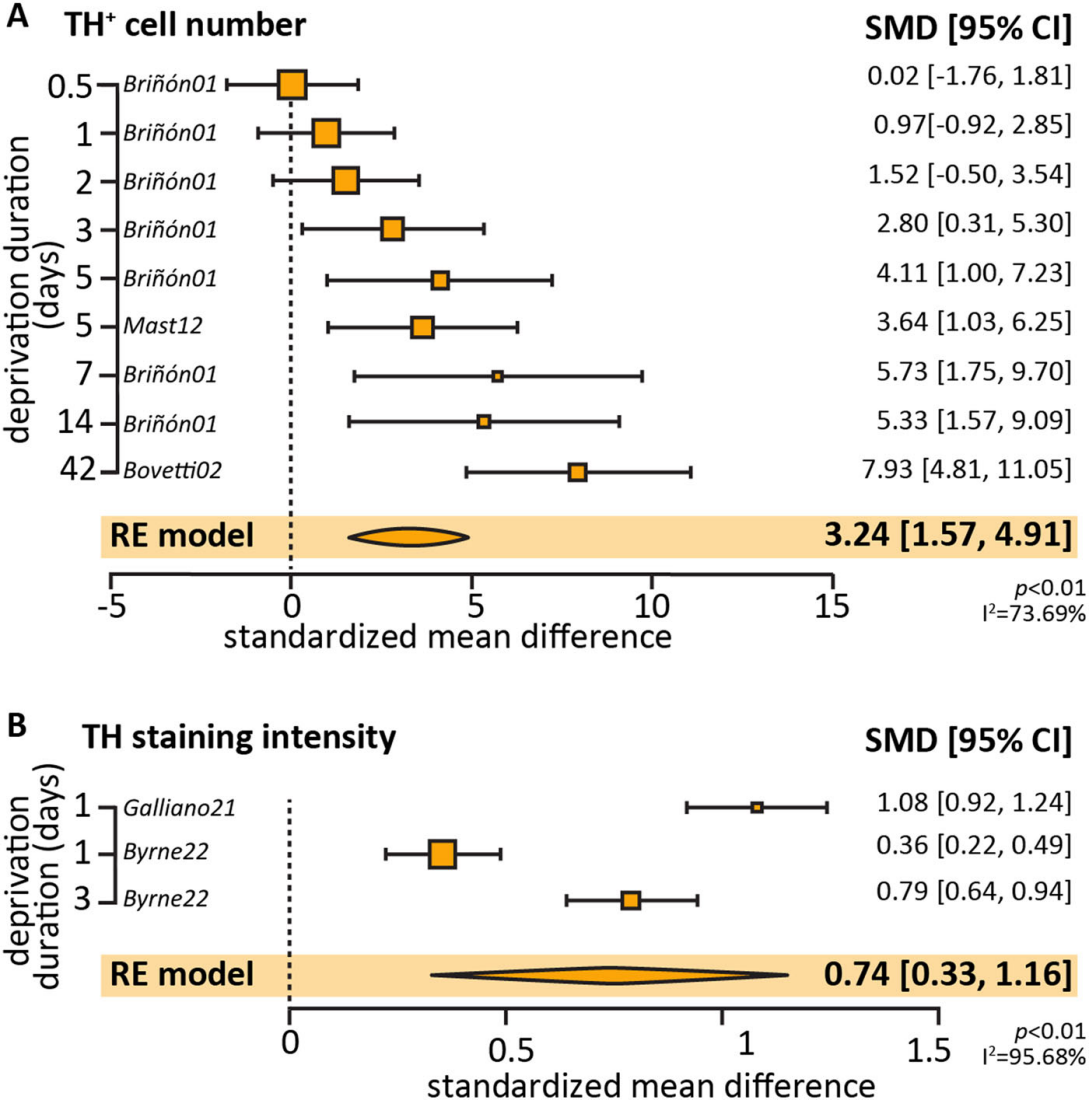
Meta-analysis of TH expression after olfactory deprivation of various durations. ***A***, Effect size of olfactory deprivation on the number of TH-positive DA neurons in the OB. Note that 7/11 data points originated from the same study (Briñón et al., 2001). ***B***, Effect size of olfactory deprivation duration on the TH staining intensity in bulbar DA neurons. Note that 2/3 data points originated from the same study (Byrne et al., 2022).

When papers defined the cell types in which they investigated deprivation-induced plasticity, they did so in different proportions across the three sensory modalities. While principal neurons have been explicitly investigated in all circuits albeit with different proportions [2/39 papers in vision (Jaepel et al., 2017; Huh et al., 2020), 3/30 papers in olfaction (Henegar and Maruniak, 1991; Wilson and Sullivan, 1995; Galliano et al., 2021), and 7/22 papers in audition (Caicedo et al., 1997; Francis and Manis, 2000; Garcia et al., 2000; Asako et al., 2005; Whiting et al., 2009; Clarkson et al., 2016; Poveda et al., 2020)], a much more diverse picture emerges when one focuses on of interneurons. Notably, while none of the vision papers investigated interneurons explicitly, most of the olfactory studies did, both historically and currently (Fig. 7*B*,*C*). Studies investigating the olfactory bulb focused on both inhibitory interneurons (23 papers, 77% of all olfaction papers) and excitatory interneurons (5 papers, 17% of all olfaction papers; see Table 1 for details). Similarly, in audition, a fair percentage of papers investigated inhibitory interneurons (5 papers, 23%) and excitatory interneurons (2 papers, 9%). Such difference was expected given the different ratios of interneurons present in these circuits, with over 80% of OB neurons being GABAergic (Shepherd, 2004). Since the turn of the century, more attention has been devoted to glial cells in all circuits (median year of investigation: 2003).

**Table 1.** Deprivation-induced plasticity

Deprivation duration	Sensory modality	Key findings
Short term	Olfaction	**Principal neurons:** decrease in immediate early gene pS6 immunoreactivity, no changes in axon initial segment structure or in intrinsic excitability (Galliano et al., 2021*) **Inhibitory interneurons:** decrease in the number of TH+ neurons in dopaminergic cells (Briñón et al., 2001), TH and c-*fos* immunoreactivity, axon initial segment length, intrinsic excitability (Galliano et al., 2021*) **Excitatory interneurons:** decrease in immediate early gene pS6 immunoreactivity, no changes in axon initial segment structure and intrinsic excitability (Galliano et al., 2021*)
	Audition	**Principal neurons:** at auditory nerve synapses increased synaptic expression of GluR2/3 (but not GluR2 or GluR4) in bushy cells, increased expression of GluR2/3 and reduced expression of GluR4 in pyramidal cells; no changes at glutamatergic synapses from parallel fibers to pyramidal cells; at inhibitory synapses decrease in the expression of GlyRa1 in bushy and pyramidal cells (Whiting et al., 2009*) **Interneurons:** decrease in calretinin immunoreactivity, transient increase in parvalbumin immunoreactivity after 6 and 12 h but then decrease to control levels by 24 h (VCN; Caicedo et al., 1997); no change in GAD65 and vGluT1 staining compared with contralateral side [VCN; (Heusinger et al., 2019)]; transient increase in c-*fos* expression in cartwheel cells (Luo et al., 1999) **Glia** Various: increased immunoreactivity in GFAP and polysialic acid; no change in GAP-43 (or in in matrix metalloproteases MMP-2 (associated re-innervation) and MMP-9 (associated with neurodegeneration) (VCN; Fredrich et al., 2013) Astrocytes: increase in astrocyte staining intensity revealed by GFAP (VCN; Janz and Illing, 2014); increased immunoreactivity in cell membrane-actin cytoskeleton linker ezrin (VCN; Fredrich et al., 2013) Microglia: increase in immune-related genes Ccl12, Csf1, and Cd44 and in activated-microglia marker Iba1 positive cell number (CN; Harris et al., 2008*); no change in CD11b staining intensity of microglia but more compact morphology and increase in immunoreactivity for different phosphorylated forms of MAPKs (VCN; Janz and Illing, 2014); increased proliferation of activated microglia (VCN; Illing et al., 2019)
	Vision	**Macroscopic and overall circuit:** no increase in apoptosis (LGN; Kawabata et al., 2003*); reduction in glucose uptake (LGN and SC; Horsburgh and McCulloch, 1991; Wang et al., 2005*); after eyelid suturing no changes in spontaneous activity or spiking temporal patterns (LGN; Linden et al., 2009*); no changes in the distribution of glutamate receptors measured with autoradiography (LGN and SC; Chalmers and McCulloch, 1991a); increased reactive oxygen species in SC after enucleation (Hernandes et al., 2010) **Glia:** increased GFAP immunoreactivity (LGN; Canady et al., 1994); increased GAT-1 and GAT-3 immunoreactivity in hypertrophied astrocytes, no change in GABA nor GAD immunoreactivity (SC; Yan and Ribak, 1998)
Medium term	Olfaction	**Macroscopic:** after surgical or chemical ablation, increase in proliferating Ki67-positive OSNs (Kikuta et al., 2015) and in proliferating cells in the OB subependymal layer (Veyrac et al., 2005); no change in OSN proliferation after nose plugging (Kikuta et al., 2015); reduced olfactory epithelium thickness (Mandairon et al., 2003) and smaller OB volume (Pothayee et al., 2017*); increased immunoreactivity for apoptosis markers caspase-3 (Mast and Fadool, 2012*) and TUNEL (Mandairon et al., 2003; Veyrac et al., 2005) but no evidence for principal cells apoptosis (Mandairon et al., 2003); increased beta secretase staining in the glomerular layer (Yan et al., 2007) **Inhibitory interneurons:** in dopaminergic cells decrease in TH immunoreactivity (Briñón et al., 2001; Lau and Murthy, 2012*; Mast and Fadool, 2012*) and reduction in DA presynaptic terminal density (Grier et al., 2016); in immature adult-born granule cells reduced spine density (Denizet et al., 2017) **Excitatory interneurons:** reduced amplitude of sIPSC and reduced levels of synaptic GAD67 puncta (Lau and Murthy, 2012*); increased amplitude of AMPAR-mediated mEPSCs (Tyler et al., 2007*) **Glia:** more reactive astrocytosis in all OB layers (Veyrac et al., 2005); increase in microglial density (Grier et al., 2016; Denizet et al., 2017); microglia more dynamic and shifted toward amoeboid morphology (Grier et al., 2016); greater percentage of neurons wrapped by microglia (Grier et al., 2016); and increased phagocytosis of adult-born granule cells (Denizet et al., 2017)
	Audition	**Macroscopic:** no change in VCN neuron number (VCN; Mostafapour et al., 2002*); no change in choline acetyltransferase activity in (VCN and DCN; Jin et al., 2005); lower glucose uptake (VCN and DCN; Tucci et al., 2001); across cell types changes in immunostaining intensity for various PKC isoforms (Garcia et al., 2000) **Excitatory neurons:** reduction of VGLUT1 immunoreactivity (VCN; Heusinger et al., 2019); reduction in the density of synaptic contact zone stained with GAP-43 (VCN; Illing and Horváth, 1995) **Inhibitory interneurons:** increase in GAD65 staining (VCN; Heusinger et al., 2019); increase in the overall fraction of inhibitory synapses and increase of GAP-43-stained nascent synapses (VCN; Illing and Horváth, 1995; Hildebrandt et al., 2011); recovery to control level in calretinin and increase in parvalbumin and calbindin immunoreactivity VCN (Caicedo et al., 1997; Förster and Illing, 2000*) **Glia:** increase in GFAP, GAP-43, PSA, and MMP-2 staining intensity (VCN; Fredrich et al., 2013; Janz and Illing, 2014); increase in p-38 and microglial immunoreactivity (CD11b; VCN; Janz and Illing, 2014); increase in calbindin-positive astrocytes (VCN; Förster and Illing, 2000*); and increased expression in astrocytes of Ncam and Agg (VCN and DCN; Heusinger et al., 2019); decrease/plateau of ezrin and MMP-9 (VCN; Fredrich et al., 2013) and of p-ERK1/2 immunoreactivity (VCN; Janz and Illing, 2014)
	Vision	**Macroscopic:** reduced SC thickness (Yan and Ribak, 1998); increased number of LGN neurons with darkly stained perikaryon and pyknosis (apoptosis indicator; Gonzalez et al., 2005), but no changes detected with ApopTag (Kawabata et al., 2003*); reduced glucose uptake in LGN (Zilles et al., 1989); in LGN decrease in immediate early genes expression NGFI-A (Giraldi-Guimarães and Mendez-Otero, 2005) and c-*fos* (Gonzalez et al., 2005). No change in cAMP response element binding protein in SC (Vierci et al., 2013*), neurokinin-1, GABAa, and serotonin-2–binding sites (SC; Boulenguez et al., 1993); reduced nitric oxide synthase labeling in SC (Batista et al., 2003); increased reactive oxygen species in SC after enucleation (Hernandes et al., 2010); rearrangement of the retinocollicular topography after partial optic nerve crush (Kreutz et al., 2004); more calretinin-positive cells in SC contralateral to enucleation (Arai et al., 1993); reduced 5-HT1 receptor binding in SC and dLGN (Chalmers and McCulloch, 1991b); increased enkephalin staining in SC (Miguel-Hidalgo et al., 1991, 1990) **Excitatory pathways:** increase in maximum AMPAR-mediated EPSC amplitudes and decrease in single-fiber AMPAR-mediated EPSC amplitudes at retinogeniculate synapse (LGN; Hooks and Chen, 2008*); increased stargazing phosphorylation at retinogeniculate synapse (LGN; Louros et al., 2014*); LGN TS neurons shift responsiveness from monocular to binocular (Jaepel et al., 2017) **Inhibitory neurons:** weaker calbindin immunoreactivity and increased number of parvalbumin-positive neurons in LGN (Gonzalez et al., 2005); increased number of calretinin-positive in SC (Lane et al., 1996) **Glia:** increased number of GFAP immunoreactive astrocytes in LGN (Gonzalez et al., 2005); substantial glia activation after partial optic nerve crush (Kreutz et al., 2004)
Long term	Olfaction	**Macroscopic** Proliferation: reduction in Ki67-positive proliferating cells (Kikuta et al., 2015), slower migration of neuroblast along the RMS and integration in the OB (Pothayee et al., 2017*) Protein expression: no significant change in expression of neurogranin (postsynaptic calmodulin binding protein; Gribaudo et al., 2012*) Size: with nose plug smaller OB volume after 3 weeks (Pothayee et al., 2017*); no changes in OSN histology after 4 weeks (Kikuta et al., 2015); with chemical or surgical ablation decrease in OB weight after 1–6 months (Maruniak et al., 1989); smaller OB but no change in cell number after 20 d (Hamilton et al., 2008); epithelium thickness, OSN histology and apoptosis marker are back to control level after 28 d (Mandairon et al., 2003; Kikuta et al., 2015) **Principal neurons:** no change in M/TC number after 5 months occlusion (Henegar and Maruniak, 1991) but M/TC apoptosis after 30 d occlusion (Fiske and Brunjes, 2001*); no change in granule cells mediated IPSCs, decreased odor discrimination and lower response threshold, no changes in spontaneous activity (Wilson and Wood, 1992*; Wilson and Sullivan, 1995*) **Excitatory interneurons:** broadening of ETCs tuning curves (Marks et al., 2006*) **Inhibitory interneurons** Various: no change in GAD immunoreactivity after 5–9 weeks deprivation (Kosaka et al., 1987*), decrease of GAD67 but not GAD65 protein levels after 14 d deprivation (Parrish-Aungst et al., 2011), no change in apoptosis in calbindin and calretinin cells (Sawada et al., 2011); decrease in newborn cell density in the glomerular layer (Bovetti et al., 2009; Sawada et al., 2011); in newly generated cells no difference in the expression of GAD67, calretinin, calbindin (Bovetti et al., 2009) EPL interneurons: no difference in parvalbumin cell number and apoptosis (Hamilton et al., 2008; Sawada et al., 2011); reduced GluR1 and GAD65 immunoreactivity (Hamilton et al., 2008) Dopaminergic cells: decreased TH immunoreactivity and/or decreased TH-positive cell number and/or increased apoptosis (Kosaka et al., 1987; Wilson and Sullivan, 1995; Philpot et al., 1997; Yan et al., 2007; Parrish-Aungst et al., 2011; Sawada et al., 2011; Bovetti et al., 2013, 2009; Pérez-Revuelta et al., 2020); decrease in TH-positive newborn cells (Bovetti et al., 2009); decrease in dopamine and DOPAC levels, no change to NE or DHPG (Philpot et al., 1997) Granule cells: reduction of synaptic puncta in the internal plexiform layer, but not external plexiform layer (Cummings and Belluscio, 2010); increased secretagogin expression (Pérez-Revuelta et al., 2020); decreased number and soma size (Henegar and Maruniak, 1991); decrease in adult born cell number and significant increase in apoptosis after 4 weeks (Yamaguchi and Mori, 2005); in newly generated cells reduced density of glutamatergic inputs and of output synapses, while in resident cells increase of synaptic inputs on the proximal dendrites (Kelsch et al., 2009); increased GrCs apoptosis after 30 d occlusion (Fiske and Brunjes, 2001*)
	Audition	**Macroscopic** Size: reduction in VCN area and neuron density (Yuan et al., 2014); decrease in number of GAP-43 nascent synapses (VCN; Illing and Horváth, 1995) Protein expression: increased choline acetyltransferase activity after 1 month and returns to control levels after 2 months (VCN; Asako et al., 2005; Jin et al., 2005); increased expression of Kv1.1 and Kv3.1b in the VCN (Poveda et al., 2020); reduction in the fraction of inhibitory synapses but no changes in overall synaptic contact zone in VCN after 70 d (Hildebrandt et al., 2011) **Excitatory pathways:** Decrease in VGLUT1 expression in auditory nerve terminals across both nuclei (Yuan et al., 2014; Heeringa et al., 2016*); VGLUT2 increases in the interstitial region and fusiform layer of DCN and AVCN (Heeringa et al., 2016*); in GluA3-KO mice auditory brainstem responses recovered more slowly than in wild-type animals following 10 d of ear plugging (García-Hernández et al., 2017*) **Principal neurons:** decreased glycine immunoreactive puncta in pyramidal cells and bushy cells (Asako et al., 2005); increased of postsynaptic density thickness and upregulation of GluA3 subunit expression on bushy cells (Clarkson et al., 2016*); in VCN type I stellate and bushy cells are more depolarized resting potential and have smaller action potentials, while there are no changes in repetitive firing of type II bushy cells (Francis and Manis, 2000*) **Inhibitory neurons:** decreased glycine immunoreactive puncta in both types of VCN stellate cells (Asako et al., 2005); no changes in PKC gamma isoform 28 d post deafferentiation (Garcia et al., 2000) **Glia:** more calbindin-positive astrocytes then after 30 d number starts to decline (VCN; Förster and Illing, 2000*)
	Vision	**Macroscopic** Size: increase in apoptotic cells number in the dLGN after 12 d but no change in the vLGN (Kawabata et al., 2003*); decrease in volume and thickness of the superficial layers of the SC (García del Caño et al., 2002; Gerrikagoitia et al., 2002, 2001); increased staining intensity of V1 terminals in the upper half of the superficial SC (García del Caño et al., 2002); increased density of serotonin immunoreactive fibers in superficial SC and LGN (Rhoades et al., 1990); increased density of locus ceruleus to LGN projections if deprived at P30, no change if deprived at P60 (Nakamura et al., 1984*); no change in noradrenergic fiber inputs nor beta-adrenergic receptors binding after in the SC after 1 month deprivation in P365 animals (Muguruma et al., 1997); 2 weeks after nerve crush increase in non-myelinated fasciculated axons (Kreutz et al., 2004) Activity: reduced cytochrome oxidase activity in LGN and SC (Land, 1987; Sukekawa, 1987); 8 weeks after deprivation vLGN cytochrome oxidase activity recovered to control level while activity levels in LGN and SC remained lower, and 12 weeks after deprivation only SC had a significantly lower cytochrome oxidase activity (Sukekawa, 1987); reduction in glucose uptake in SC and/or LGN (Horsburgh and McCulloch, 1991; Thurlow and Cooper, 1991); increased binding of kainate, reduced binding of AMPA after 20 d (Chalmers and McCulloch, 1991a); increase in choline acetyltransferase activity (SC; Gerrikagoitia et al., 2002); 15 d after enucleation reactive oxygen species in SC have been reported as increased by (Hernandes et al., 2010) and reduced by (Batista et al., 2003) Protein expression: increased substance-P immunoreactivity (LGN; Senba and Miguell-Hidalgo, 1993); increased enkephalin staining in SC (Miguel-Hidalgo et al., 1990); increased expression of GAP-43 after 1 week and back to control levels after 2–3 weeks (SC; Mendonça et al., 2010*); increased number thymosin beta 4-positive neurons and with longer and more complexly branched neurites (SC; Paulussen and Arckens, 2012) **Principal neurons:** increase in the proportion of multi-innervated neurons and larger AMPAR-mediated EPSCs (dLGN; Narushima et al., 2016*); decrease in single-fiber AMPAR-mediated EPSC amplitudes at retinogeniculate synapse (LGN; Hooks and Chen, 2008*; Narushima et al., 2016*); no changes in the receptive fields in neurons of superficial SC cells (Carrasco and Pallas, 2006); disruptions in binocular integration in dLGN (Huh et al., 2020*) **Interneurons:** fewer parvalbumin neurons in the LGN (Gonzalez et al., 2006) and more parvalbumin neurons in the superficial SC (Lane et al., 1996); decrease in calbindin expression in vLGN (Gonzalez et al., 2006) but no change in SC (Lane et al., 1996); reduced GABAA receptor binding 20 d after enucleation in dLGN (Chalmers and McCulloch, 1991b) **Glia:** higher GFAP staining after 3 weeks with thick and branched processes visible (LGN; Canady et al., 1994); decrease in number of GFAP immunoreactive astrocytes in LGN after 12–48 weeks (Gonzalez et al., 2006)

Summary of findings across the three senses divided by deprivation duration: short term, day or less; medium term, between 1 d and 1 week; long term, over a week. All 91 papers included in the study are cited at least once, and starred references indicate studies which used juvenile animals between weaning and the 40th postnatal day.

### Effects of sensory deprivation-induced plasticity

Our objective was to assess the consistency, directionality, and comparability of the deprivation-induced effects across systems in a quantitative manner, performing a meta-analysis of the literature. Unfortunately, this proved impossible given the huge variability in the deprivation induction method (Fig. 5), experimental techniques employed to readout the deprivation-induced plasticity (Fig. 6), as well as severity of deprivation (i.e., lesion or reversible stimulus removal; Fig. 7) and completeness of the reporting.

Reliable activity-dependent molecular markers can be used to validate the success of a deprivation method. However, in vision and audition, such a pronounced and stereotypical change has not been systematically described, albeit calcium-binding proteins and immediate early genes are potential candidates (Philpot et al., 1997; Barmack and Qian, 2002; Gonzalez-Perez et al., 2018). In olfaction, reduced expression of tyrosine hydroxylase (TH) has been traditionally used to confirm that the nose plugging or cauterization had an effect (Baker et al., 1983). As such, TH RNA and/or protein expression is the most reported plasticity readout across the literature. Using the same search terms to extend the publication date to March 2023 to increase our paper pool to a statistically meaningful size (Andrade, 2020), we collated 11 papers reporting TH changes using immunofluorescence, and we attempted to conduct a meta-analysis to assess the overall effect of olfactory deprivation on TH expression. While all studies showed a decrease in TH, we wanted to see if the change was dependent or modulated by deprivation duration. Out of the 11 papers, the ones with sufficient statistical reporting allowing for meta-analysis (5 papers) were split into ones that used TH-positive cell density (9 experiments across 3 papers) versus those based on TH cell fluorescence (3 experiments across 2 papers) to report TH changes. The overall measurement of standardized mean difference was significant for both measurements, 3.24 (random-effects meta-analysis, *p* = 0.0001) for density and 0.74 (random-effects meta-analysis, *p* = 0.0004) for the staining intensity, demonstrating that olfactory deprivation is correlated with a decline in TH expression. However, upon further analysis, the test for heterogeneity in each case (*I*^2^ = 73.69%, *p* = 0.0003 and *I*^2^ = 95.68%, *p* < 0.0001) shows that we cannot assume that there is a singular effect size for the population represented by these studies. When occlusion duration was added as a modulator, the amount of heterogeneity unaccounted for by occlusion time was very high in studies investigating TH by fluorescence. This was lower for studies of TH density analysis (*I*^2^ = 23.07%, *p* = 0.2486), suggesting a more homogeneous underlying effect size once occlusion duration was accounted for. The low number of experiments and papers entered in the meta-analysis precludes any conclusions from being drawn as the number of studies falls below the threshold of minimum of 10 experiments used by meta-analyses in the field (Andrade, 2020).

Given the impossibility to perform quantitative meta-analysis on the entire dataset, we attempted to summarize and correlate these results qualitatively by grouping the findings using the only descriptor present in every study: the overall duration of the deprivation, which we divided in short term (1 d or less), medium term (up to a week), and long term (over a week). This grouping, albeit arbitrary, reflects physiologically relevant scenarios of changes in sensory inputs. Short-term deprivation reflected transient and mild diseases involving sensory organs, for example, temporary hearing loss from noise overexposure (Syka and Rybalko, 2000), loss of smell from mild colds (Fokkens et al., 2012), and transient visual loss (Pula et al., 2016). Medium-term deprivation reflects longer albeit still temporary diseases, such as acute ear infection (Worrall, 2007) and anosmia associated with COVID-19 (Santos et al., 2021). Long-term deprivation reflects severe, more permanent forms of sensory deprivation, such as presbycusis (age-related hearing loss; Gold and Bajo, 2014), long COVID-19 (Park et al., 2022), and diabetic retinopathy (Stitt et al., 2016). Where possible we tried to group findings by cell-type (Fig. 7) and broad subregion (e.g., dorsal LGN/CN vs ventral LGN/CN). When cell type specificity was not reported, the results were divided into a few broad themes, namely, macroscopic size, protein expression, activity, and proliferation. Using this structure, a few central commonalties emerged and are summarized below and in Table 1.

### Common mechanisms of plasticity: short-term deprivation produces decreased activity and disinhibition

After short-term deprivation, defined as 24 h or less, the effect most consistently investigated across all three senses was changed in overall activity levels. Despite using different proxies, a reduction in neuronal metabolic activity in the early brain circuits receiving input from the deprived eye/ear/nostril was found across all studies. In the visual system, this is manifested as a reduction in glucose uptake in the LGN and SC (Horsburgh and McCulloch, 1991; Wang et al., 2005). Similarly, a decrease in the immunoreactivity of the activity early gene c-*fos* was observed in the olfactory (Briñón et al., 2001; Galliano et al., 2021) and auditory systems (Luo et al., 1999, p. 199). This rapid decrease in activity is in alignment with that found in primary visual and auditory cortices (Feldman, 2009; Gold and Bajo, 2014).

Deprivation-induced homeostasis can be achieved, at least transiently, by balancing the changes in the excitatory and inhibitory pathways, and it has been proposed that rapid disinhibition mediated by downregulated inhibitory networks precedes excitatory plasticity (Barnes et al., 2015; Gainey and Feldman, 2017). The papers that defined cell types and specifically investigated inhibition reported findings which were consistent with decreased inhibition: GABAergic and glycinergic mechanisms are downregulated following brief sensory deprivation (see Table 1 for details). In the auditory system, GlyRα1 postsynaptic density in bushy and fusiform cells was decreased (Whiting et al., 2009). In the olfactory system, the dopaminergic inhibitory interneurons downregulate TH expression, reduce their intrinsic excitability, and shorten the axon initial segment (Galliano et al., 2021). In visual areas, immunoreactivity of GABA transporters (GAT-1 and GAT-3) in hypertrophied astrocytes in the deafferented SC was increased, suggesting an increased uptake of GABA (Yan and Ribak, 1998). This early disinhibition of the deprived system, achieved via different mechanisms in each circuit, also occurs in higher areas and has been extensively reviewed in the auditory, visual, and somatosensory cortices (Gold and Bajo, 2014; Li et al., 2014; Gainey and Feldman, 2017).

Conversely, alterations in excitatory signaling following 1-day-long deprivation were less clear cut. In olfaction and vision, studies which investigated glutamatergic function found no differences after brief deprivation. In vision, this was investigated using autoradiography, and no changes were found in the SC or dLGN for AMPA, NMDA, and kainate receptors expression (Chalmers and McCulloch, 1991a). In the olfactory system, while both the glutamatergic principal neurons and interneurons downregulated the expression of immediate early genes and activity markers, they did not modulate their intrinsic excitability or their axon initial segment morphology (Galliano et al., 2021). In the auditory system, however, there is a highly specific upregulation of AMPA receptor subunits at the auditory nerve to principal neuron synapse (GluR3 upregulated at bushy cell and fusiform synapse, GluR4 downregulated at fusiform synapse; Whiting et al., 2009). When combined with concomitant changes in inhibition, the overall functional effect of these changes on circuit homeostasis remains to be elucidated.

In contrast to the other two senses, papers in audition mostly focused on short deprivation, with almost half of all papers investigating 1 d or <1 d deprivations (9/22; Fig. 5*B*) and glial responses. An increase in glial activation was found across different studies using varied experimental methods and included increases in immune-related genes (Harris et al., 2008), activation (Harris et al., 2008; Janz and Illing, 2014), and proliferation (Illing et al., 2019) of microglia, as well as increases in staining for astrocyte markers (Janz and Illing, 2014). These findings are largely consistent with an increased glial activity in the cochlear nuclei, but given the lack of similar investigations in vision and olfaction, it remains unclear whether this is a unique feature of the early auditory circuits or, like decrease in activity and disinhibition, a common response to brief sensory deprivation.

### Common mechanisms of plasticity: medium-term deprivation is reflected by an increased involvement of glia and synaptic remodeling

After mid-term deprivation, defined as lasting between 1 d and a week, we found activity reductions (e.g., in glucose uptake after visual deprivation) which were consistent between animals deprived at juvenile or adult stages (Zilles et al., 1989; Wang et al., 2005). Alongside continued decreases in activity, the most prominent changes were changes in synaptic properties and glial activation. Across all three sensory modalities, multiple studies reported functional and structural changes at the synapses formed by the cranial nerve axon terminals, suggesting an overall change to circuit-level excitation/inhibition (E/I) balance.

In the olfactory system studies, investigations did not focus on presynaptic remodeling but rather on the postsynaptic properties of the various interneuron subtypes innervated by the olfactory nerve directly or via multisynapse loops. Functional changes have been found in juvenile mice at the excitatory interneurons (external tufted cells, ETCs), where deprivation decreased the amplitude of spontaneous inhibitory postsynaptic currents (Lau and Murthy, 2012), as well as increased quantal glutamatergic (AMPA-mediated) postsynaptic currents (Tyler et al., 2007). Structurally, a reduction of spine density was found in the inhibitory interneurons granule cells (Denizet et al., 2017) as well as GAD67 puncta in ETCs (Lau and Murthy, 2012). Since ETCs modulate both inhibitory interneurons and excitatory principal neurons in the OB, the overall effect of these changes on the whole-circuit output remains unclear but seems to suggest modifications in the E/I balance.

In the visual system, only synapses formed by the optic nerve onto principal neurons in the dorsal LGN were studied after medium-term sensory deprivation. Thalamocortical neurons in the dLGN of juvenile animals had smaller single-fiber AMPA-mediated postsynaptic currents at the retinogeniculate synapse (Hooks and Chen, 2008; Narushima et al., 2016), indicating weakening of individual glutamatergic afferents following deprivation. However, this was counterbalanced by a concomitant increase in excitatory postsynaptic current (EPSC) amplitudes, indicating an increase in the number and/or strength of retinogeniculate synapses (Hooks and Chen, 2008). Structurally, there was an increase in the phosphorylation of stargazin, a transmembrane AMPAR trafficking protein in the LGN involved in synaptic scaling (Louros et al., 2014). Overall, the evidence indicates that visual deprivation induces synaptic remodeling at the excitatory retinogeniculate synapse in the dLGN of juvenile rodents after medium-term deprivation.

In the auditory system, the most prominent synaptic effect of 1 week deprivation was the overall macroscopic decrease in the number of synaptic contact zones in the anterior VCN (Hildebrandt et al., 2011). Along similar lines, a significant reduction in VGLUT1 staining was found in the VCN (Heusinger et al., 2019). Interestingly, inhibitory synapses decreased in number less markedly than excitatory ones (Hildebrandt et al., 2011). Taken together with the findings of rapid disinhibition described above, this could suggest a period of transient overinhibition following an early disinhibition phase of the VCN after cochlear deafferentation.

In addition to changes in synaptic transmission, an increase in glial proliferation and activation was found consistently across all senses. In the olfactory system, significant increases in the density and activation of microglia were seen in all papers which investigated this phenomenon (Grier et al., 2016; Denizet et al., 2017). The increased activation of microglia wasevident both in the morphology (shown as fewer primary microglial processes and shift toward hypertrophied morphology; Grier et al., 2016) and immunoreactivity staining with specific marker for activated microglia CD68 (Denizet et al., 2017). Moreover, a significant increase in reactive astrocytes was reported in all OB layers (Veyrac et al., 2005), as well as in the visual system (although here it was not a formal strand of investigation; Gonzalez et al., 2005). In the auditory system in addition to increases in microglial and astrocytic immunoreactivity in both juvenile and adult animals (Förster and Illing, 2000; Janz and Illing, 2014), glial activation has been linked to synaptogenesis in both the VCN and DCN (Fredrich et al., 2013; Heusinger et al., 2019). During the first 3 d of deprivation, astrocytes increased production of neurocan, aggrecan, and MMP9, which are key for synapse stabilization (neurocan and aggrecan; Asher et al., 2000; Rowlands et al., 2018) and the induction of synaptic plasticity via ECM remodeling (MMP9; Huntley, 2012). Then, between 3 and 7 d of deprivation, expression of these proteins decreased and was replaced by PSA-NCAM and MMP2. Both are strongly associated with structural plasticity, MMP2 via neurocan digestion and PSA-NCAM by mediating synaptogenic interactions between neurons and astrocytes (Hillen et al., 2018; Akol et al., 2022). This astrocytic biphasic response and its potential role in synaptic remodeling warrants further research in the visual and olfactory regions.

### Common mechanisms of plasticity: long-term deprivation

Given the adopted definition of long-term deprivation as any period spanning between a week and a year, we found that the effect on experience-dependent plasticity on the different cell types was more heterogeneous than for shorter-lasting deprivations. Variability notwithstanding, remarkably consistent macroscopic changes and apoptosis were found across all three senses in both juvenile and fully adult animals. In olfaction, this was reflected by a smaller overall OB volume and weight (Maruniak et al., 1989; Hamilton et al., 2008; Pothayee et al., 2017) as well as in a reduction of external plexiform layer thickness (Hamilton et al., 2008) and glomeruli volume (Cummings and Belluscio, 2010) and in increased cell death in the mitral and granule cell layers (Fiske and Brunjes, 2001). Similarly, increased apoptosis was seen in the dLGN (Sukekawa, 1987) and reduction in volume was seen in the stratum zonale, stratum griseum superficiale, and stratum opticum regions of the SC (García del Caño et al., 2002; Gerrikagoitia et al., 2002). In audition, there was a marked decrease in VCN area and neuron quantity (Yuan et al., 2014). This contrasts with short-term deprivation, where macroscopic structural changes were scarcely investigated. Among our included papers, only one study reported no difference in apoptotic neurons between the unperturbed and deprived side (Kawabata et al., 2003).

In the olfactory system, these macroscopic changes can manifest in a rather unique way, namely, in cellular turnover. Neural progenitors are produced in the subventricular zone and migrate along the rostral migratory stream (RMS). They arrive at the subependymal layer of the OB, where they differentiate into glomerular interneurons or granule cells (Carleton et al., 2003). The experience-dependent nature of adult neurogenesis has been widely researched (reviewed in Bonzano et al., 2016), and it is well known that olfactory deprivation and enrichment can decrease or increase the survival of adult-born neurons, respectively. The findings of this review were in alignment with the literature, where after medium- to long-term deprivation, neurogenesis was reduced at each level of the neurogenic process. After 2–3 weeks deprivation, there was a reduction in the integration of neuroblasts into the OB, as well as slower migration along the RMS in juvenile animals (Pothayee et al., 2017). In the glomerular layer of adult rodents, a decrease of newborn periglomerular cells was found after 28 d (Sawada et al., 2011) and 42 d deprivation (Bovetti et al., 2009). This is mirrored by an increase in cell apoptosis in both the periglomerular (Sawada et al., 2011) and granule cells (Yamaguchi and Mori, 2005). Remarkably, this experience-dependent neurogenesis is found to be very specific to the dopaminergic population at the glomerular layer, as the calbindin and calretinin glomerular interneuron populations remained unchanged after deprivation (Sawada et al., 2011). Unfortunately, no papers investigated neurogenesis in the context of shorter deprivations, so we cannot correlate these findings with the deprivation duration.

According to theoretical frameworks and working models in the homeostatic plasticity field, the expectation is to see prominent changes in excitatory networks with a long-lasting sensory deprivation as the circuit stabilizes to a new set point (Gainey and Feldman, 2017). While no papers focused on the early visual circuits’ principal neurons responses to long-term deprivation, changes at the excitatory synapse between incoming nerve and principal neurons were seen at bushy cells in the cochlear nuclei. Ten days of auditory deprivation using earplugs was correlated with a decrease of the presynaptic marker VGlut immunoreactivity and size of synaptic vesicles in juvenile rats (Clarkson et al., 2016). A similar VGlut decrease was observed in the VCN of adult rats 3–14 d after cochlear ablation, suggesting a robust effect in both manipulations (Heusinger et al., 2019). Postsynaptically, GluA2/3 expression was upregulated, while GluA2 and GluA4 remained unchanged (Clarkson et al., 2016)—an effect found also after 1 d deprivation in P30 rats (Whiting et al., 2009). In the olfactory system, the systematic search only returned one paper which investigated mitral/tufted cells after long-term deprivation in juvenile rats (Wilson and Sullivan, 1995). After 60 d deprivation, while there were no changes in their spontaneous activity rates, the number of mitral/tufted cells responding to more than one odor was increased. In addition, significantly more cells in the deprived bulb responded to specific odors presented at higher intensities, suggesting a possible decrease in odor discrimination coupled with increase in responsiveness. Conversely, in a very recent paper published after the cutoff date of our systematic search (George et al., 2022), the authors found a shortening in the mitral cell axon initial segment as well as spiking frequency in adult mice unilaterally occluded for 30 d compared with the un-occluded bulb, indicating potential for decrease in intrinsic excitability at OB principal neurons after longer deprivation times. The slight discrepancy in mitral/tufted cell firing rate after long deprivation found by Wilson and Sullivan (1995) and George et al. (2022) could be attributable to various methodological differences, such as occlusion times (30 d vs 60 d, respectively), animal model (P30 rats vs P60 mice, respectively), as well as experimental methodology (in vivo electrophysiology vs acute slice electrophysiology, respectively).

In addition to changes at the principal neurons, deprivation on a longer time scale also sees a decrease in glial activation, which was investigated in vision and audition. In vision, the number of immunoreactive astrocytes significantly decreased 12–48 weeks after enucleation (Gonzalez et al., 2006). In audition, the number of calbindin-positive astrocytes started to decrease 30 d after cochlear lesion (Förster and Illing, 2000).

## Discussion

In this study we employed systematic and meta-analysis methods to investigate the effects of sensory deprivation of various durations at the homologous regions receiving the cranial nerve input of three sensory modalities—vision, olfaction, and audition—in rodents. Our analysis returned large disparities across sensory modalities in publication trends, experimental methodologies employed, as well as focus of the research. However, despite such methodological and reporting differences, a few shared findings describing adaptive responses and compensatory mechanisms to deprivation can be extrapolated.

### Profound differences in micro circuitry, experimental protocols, plasticity readouts, and reporting make comparisons across the three sensory modalities challenging

The three senses differ in their micro circuitry, as well as in the degree to which it has been characterized (Fig. 1). The lack of studies defining cell types and investigating interneurons in vision could be partially attributed to the incomplete characterization of the LGN, where cell identification is challenging by its lack of overt lamination (Duménieu et al., 2021). Indeed, while the OB is a highly inhibitory circuit where the ratio of GABAergic interneurons to excitatory neurons is much higher than the other parts of the brain (Shepherd, 2004), visual structures such as the LGN are composed of mainly excitatory projection neurons (Leist et al., 2016).

The main experimental methods used to induce and interrogate plasticity also differed widely across the three senses. While all sensory deprivation approaches are long-established (Gudden, 1870; Coppola, 2012), visual deprivation via monocular enucleation and eyelid suturing garnered prominence and widespread adoption after the seminal work of Hubel and Wiesel (1963). Thus, many vision papers in our list were older and tended to use this surgical technique, while papers focusing on olfactory and audition deprivation were more recent and more commonly employ the well-established reversible procedure of nose and ear plugging.

Of note, contrary to rods, cones, and hair cells, the olfactory sensory neurons are capable of regenerating throughout the life of the animal (Cheetham et al., 2016). This poses an important difference between senses. While deprivation by deafferentation (direct lesion to the peripheral sensory neurons) and by sensory deprivation (removal of sensory stimulation while leaving the anatomy of the circuit intact) has largely similar effects in the visual and auditory systems as both are incapable of regenerating, the effects in the olfactory system can be divided into adaptive plasticity (elicited by sensory deprivation, e.g. nose plug, and within the remit of this study) and regenerative plasticity (elicited by ablation of OSNs using olfactotoxic drugs such as methimazole and dichlobenil; Bergman et al., 2002; Cummings and Belluscio, 2008).

Adding onto the anatomical and procedural discrepancies across the senses, a profound lack of consistency in reporting precluded the possibility of a systematic meta-analysis. Furthermore, the findings of this study are heavily subject to publication bias, as the lack of findings in a particular area does not necessarily denote a negative finding. For example, we found an abundance of studies demonstrating the role of glia in regulating the cochlear nuclei response to short auditory deprivation, but it remains unclear whether this is because glial plasticity was never investigated in olfaction and vision or rather that it was investigated, found absent, and not published.

Moreover, by focusing on postweaning sensory deprivation, our analysis included studies involving both juvenile rodents and animals reported either as older than 40 d or simply as “adult.” This potentially introduces confounding factors associated with developmentally regulated plasticity, and it is particularly noteworthy given the heterogeneity of “critical” or “sensitive” periods across different senses and regions. For example, rodents are born deaf and blind but with a functional sense of smell (Neff, 2019). The olfactory map is then shaped during early postnatal development, and the existence of a postweaning, late critical period is still debated (Poo and Isaacson, 2007; Inoue et al., 2021). Conversely, in rodents eye, opening and hearing onset only occur approximately postnatal day 10 and “late” critical periods, which have been extensively characterized in the respective cortices, and can extend as late as the fifth postnatal week (Hooks and Chen, 2007; Nakamura et al., 2020). Drawing direct comparisons between sensory modalities in juvenile animals is therefore complex. Just over a fourth of the studies in our dataset involved rodents younger than 40 d (Fig. 4*B*), and the overall trends of deprivation-induced plasticity reported in these studies align with the findings from adult-only studies (e.g., activity reduction, glia activation, early disinhibition, long-term apoptosis). Yet, our analysis may have overemphasized plasticity effects in vision and/or audition more than olfaction, especially regarding short- and medium-term remodeling of excitatory synapses. A dedicated study focusing on late critical period plasticity in precortical areas, along with more standardized reporting of the age and developmental milestones of the animals across all experiments, is needed.

### Despite procedural differences, the three early sensory areas share deprivation-dependent plasticity motifs

**Figure 9.**
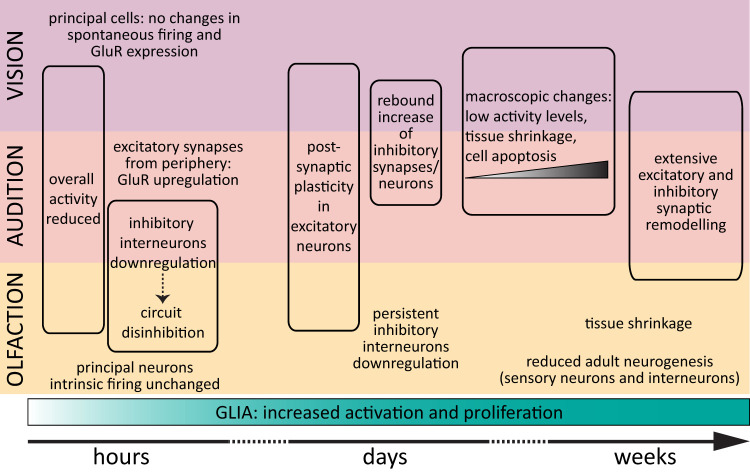
Deprivation-induced plasticity. Graphical representation of the cellular and circuit effects of sensory deprivations of increasing durations in early visual, auditory, and olfactory areas. See Table 1 for details and references.

With the caveat that the short–medium–long deprivation duration classification is partially arbitrary, albeit based on clinical evidence (Syka and Rybalko, 2000; Worrall, 2007; Fokkens et al., 2012; Gold and Bajo, 2014; Pula et al., 2016; Stitt et al., 2016; Santos et al., 2021; Park et al., 2022), we found four consistent plasticity responses across the senses (Fig. 9). First, for sensory deprivation lasting up to 24 h, the overall neuronal metabolic activity is consistently reduced. In addition, the dampening of inhibitory interneuron activity was consistently reported in olfaction and audition (but not investigated in vision), suggesting fast-acting circuit disinhibition. This is in line with data from sensory cortices and the working hypothesis that excitatory principal neuron plasticity is preceded by a depression of inhibitory interneurons (Barnes et al., 2015; Gainey and Feldman, 2017). Second, in these early sensory areas, long-term and/or permanent sensory deprivations result in more drastic changes involving macroscopic changes to the circuit architecture which manifest as tissue shrinkage, cell apoptosis, and reduced adult neurogenesis in the olfactory system. Third, there is consistent evidence across all three early sensory areas and all deprivation durations for glial activation and proliferation. This highlights the prominent role of glia in shaping neuronal plasticity (Sancho et al., 2021). Fourth, although less clear-cut, from medium-term sensory loss onward, there is a strong indication of synaptic remodeling and changes in E/I balance. The fact that these four effects were revealed from the systematic search despite profound differences in circuit architecture, methodology, and readouts is perhaps an indication that these plasticity motifs are robust and conserved.

It remains unclear whether these findings can be generalized to other areas, though the time course and multiplicity of mechanisms of deprivation-induced plasticity in precortical regions found in this study align with those found in the cortex and fit with the canonical theories of homeostatic plasticity (Turrigiano, 2011; Keck et al., 2017). Notably, the phenomenon that the inhibitory cells are modulated rapidly after sensory deprivation and that such changes precede adaptations in excitatory neurons has been widely reported in sensory cortices. For example, after 1 d of monocular deprivation parvalbumin-positive GABAergic basket cells in the primary visual cortex significantly reduced their firing rate and receive less synaptic excitation, while pyramidal cells remained unchanged (Hengen et al., 2013; Kuhlman et al., 2013). This rapid functional reduction of inhibitory tone is also supported by structural changes (Keck et al., 2011), and similar dynamics have been described in primary auditory (Chambers et al., 2016; Henton et al., 2023; Kumar et al., 2023) and somatosensory (Li et al., 2014; Gainey et al., 2018) cortices. Interestingly, the opposite manipulation—6 h sensory enrichment via whisker stimulation—has been shown to induce rapid downregulation of barrel cortex pyramidal cells’ excitability (Jamann et al., 2021), perhaps a neuroprotective mechanism to combat runaway excitation. This highlights a possible asymmetry in plasticity induced by sensory deprivation versus enrichment, which warrants further investigation in both cortical and subcortical regions (Keck et al., 2017).

Finally, it has been suggested that rapid disinhibition restores the system to a “juvenile” state of plasticity (Syka, 2002; Chen et al., 2011) and facilitates functional recovery by creating an environment more permissive for the induction of synaptic potentiation through long-term potentiation or spike timing-dependent plasticity in excitatory neurons (Dan and Poo, 2004; Chen et al., 2011; Gambino and Holtmaat, 2012; Li et al., 2014). However, it remains unclear whether the subcortical disinhibition we described reflects the start of functional recovery or simply a response to reduced input (Keck et al., 2011). This is of particular relevance for those wishing to use data from animal model deprivation studies to inform translational interventions in patients suffering from sudden sensory loss. Further research is required to better dissociate the mechanisms of the two responses and to understand the relationship between subcortical plasticity and its feedforward consequences in cortex.

### Recommendations

This work synthesized studies on the early stages of sensory processing in audition, vision, and olfaction, and the results revealed significant heterogeneity in deprivation method, experimental readouts, and reporting, including definition of investigated cell types. While biological restrictions of the circuits (e.g., accessibility, different animal models) may explain some of the heterogeneity, this review also highlights areas needing further research. To improve comparability within and between fields, the identification of a “gold-standard” deprivation methods and validation readouts, together with systematic reporting of statistics and deposition of raw data, are sorely needed. Furthermore, the field could better definine cell types, especially with increasing evidence, including from our results, that inhibition and excitation are differentially regulated after deprivation (Karmarkar and Buonomano, 2006; Turrigiano, 2011; Kullmann et al., 2012). Finally, since most published work focused on deprivation-induced plasticity in primary sensory cortices, it is essential to fully characterize potential effects in the precortical areas which link them to the periphery. As described above, these are not passive relays but active plasticity hotspots which send to the cortex already processed and modulated inputs. This early processing must be considered to depict the full picture of the final cortical computation and behavioral outputs.

## Conclusions

In conclusion, this systematic review and literature meta-analysis found that, notwithstanding major experimental heterogeneity in inducing and assessing deprivation-induced plasticity, the early visual, auditory, and olfactory systems largely employ shared mechanisms to change their circuit processing. Future work should strive to standardize both experimental and reporting approaches and investigate how early circuits and higher cortical areas together coordinate an appropriate adaptive response to a lack of peripheral sensory inputs.

## Data availability

https://doi.org/10.17863/CAM.104411.

## Synthesis

Reviewing Editor: Matthew Grubb, King's College London Decisions are customarily a result of the Reviewing Editor and the peer reviewers coming together and discussing their recommendations until a consensus is reached. When revisions are invited, a fact-based synthesis statement explaining their decision and outlining what is needed to prepare a revision will be listed below. The following reviewer(s) agreed to reveal their identity: C. Geoffrey Lau, Wei Wen.

Synthesis:

Both Reviewers are very positive about this manuscript. Reviewer 2 seeks clarification on an important point regarding timepoints. After consultation we agreed that this need not necessarily involve changing the search terms or the publications included in the review/analysis. Rather, there needs to be clarity about the use of the terms 'adult'/'developmental'/'critical period' etc, and the removal or explicit justification of any claims that the paper reviews exclusively adult plasticity. Essentially, more clarity is required on exactly what was reviewed/analysed, and why. Please ensure you have fully addressed both Reviewers' comments in any revised version.

Reviewer 1:

This is a timely meta-analytical review of the three major sensory modalities where scientists have worked on the sensory deprivation-dependent plasticity in these brain regions. Some findings are expectable e.g. the study of vision is older than audition and olfaction, increasing number of publications analyzing cell types, but it is still good to have this quantitatively documented. The community needs more papers like this. I am in support of publication of this article.

Minor comment

Lines 280-282: "In recent years more attention has been devoted to glial cells in all circuits (median year of investigation =2003)." I am not sure if 2003 should be classified as "recent" given the search was done in 2021.

Reviewer 2:

In this study, the authors summarize and compare past publications on circuit and neuronal plasticity induced by sensory deprivation across three unique modalities: visual, auditory, and olfactory, particularly focusing on subcortical regions implicated in these pathways. They assess the following aspects of the screened studies: publication trend over time, sex of animal used, deprivation paradigm, plasticity readouts, cell types of interest, and effects of deprivation. They then categorize studies based on the length of deprivation performed, namely short-term (<1 day), medium-term (< 1 week), and long-term (> 1 week), and show that, despite huge variabilities in experimental design, there are common features in the manifestation of neuronal/circuit plasticity under each category.

While this study has been submitted as new research, the paper reads like a miniature review. In this sense, the authors have truly done a remarkable job in providing the field with a comprehensive picture of deprivation-induced plasticity at the interface between PNS and CNS. They have cross-examined common deprivation paradigms regarding to the three different modalities, and have summarized both distinct molecular players involved in each sensory pathway and generalized mechanisms shared by all three. Based on their findings, they have also proposed recommendations for future experimental designs that could improve comparability and reproducibility. I believe the researchers in the sensory system field will find this information interesting and helpful.

My major concern for this study is whether the authors have underestimated the contribution of developmentally regulated changes to the cellular and network responses observed post sensory deprivation. The authors claim in the Introduction (line 99-101) that they have disentangled "developmentally regulated plasticity from activity-dependent plasticity in adulthood" by only including studies that use animals older than P21. First, P21 is hardly considered as entering rodent adulthood, in fact, explosive plasticity mechanisms occur during P21-45 across many brain regions. Second, this statement may be true for the olfactory system (critical period around the first postnatal week), but certainly not for the auditory and visual systems. The auditory system exhibits several sensitive developmental windows as late as P40, and the visual system critical period does not start until P21. Ample evidence in literature suggests that plasticity mechanisms are highly regulated by development during this time. Could the author explain why this factor has not been included in the meta-analysis?

## Author Response

Synthesis of Reviews:

Both Reviewers are very positive about this manuscript. Reviewer 2 seeks clarification on an important point regarding timepoints. After consultation we agreed that this need not necessarily involve changing the search terms or the publications included in the review/analysis. Rather, there needs to be clarity about the use of the terms 'adult'/'developmental'/'critical period' etc, and the removal or explicit justification of any claims that the paper reviews exclusively adult plasticity. Essentially, more clarity is required on exactly what was reviewed/analysed, and why. Please ensure you have fully addressed both Reviewers' comments in any revised version.

We thank the reviewers for their thorough examination of our manuscript and for their comments. We are pleased that our effort to quantify and summarize deprivation data across multiple modalities was acknowledged and found valuable to the field.

Below, we outline our responses and the changes we have made to manuscript, figures, and table. We are confident that these changes have enhanced the clarity and quality of our manuscript.

Reviewer 1:

This is a timely meta-analytical review of the three major sensory modalities where scientists have worked on the sensory deprivation-dependent plasticity in these brain regions. Some findings are expectable e.g. the study of vision is older than audition and olfaction, increasing number of publications analyzing cell types, but it is still good to have this quantitatively documented. The community needs more papers like this. I am in support of publication of this article.

We thank the reviewer for their support. To facilitate follow-up studies, we are preparing the entire dataset for full release under a CC BY license. To maintain anonymity, we have indicated the doi of the chosen repository in the title page, and put a placeholder in the methods section, page 5. The data will be made available as soon as possible after publication.

Minor comment

Lines 280-282: "In recent years more attention has been devoted to glial cells in all circuits (median year of investigation =2003)." I am not sure if 2003 should be classified as "recent" given the search was done in 2021.

We apologize for losing track of time and adverbs, by working with so many manuscripts from the 1980s and 1990s we mis-identified 2003 as relatively recent. We have now amended the sentence on page 10.

Reviewer 2:

In this study, the authors summarize and compare past publications on circuit and neuronal plasticity induced by sensory deprivation across three unique modalities: visual, auditory, and olfactory, particularly focusing on subcortical regions implicated in these pathways. They assess the following aspects of the screened studies: publication trend over time, sex of animal used, deprivation paradigm, plasticity readouts, cell types of interest, and effects of deprivation. They then categorize studies based on the length of deprivation performed, namely short-term (<1 day), medium-term (< 1 week), and long-term (> 1 week), and show that, despite huge variabilities in experimental design, there are common features in the manifestation of neuronal/circuit plasticity under each category.

While this study has been submitted as new research, the paper reads like a miniature review. In this sense, the authors have truly done a remarkable job in providing the field with a comprehensive picture of deprivation-induced plasticity at the interface between PNS and CNS. They have cross-examined common deprivation paradigms regarding to the three different modalities, and have summarized both distinct molecular players involved in each sensory pathway and generalized mechanisms shared by all three. Based on their findings, they have also proposed recommendations for future experimental designs that could improve comparability and reproducibility. I believe the researchers in the sensory system field will find this information interesting and helpful.

We thank the reviewer for their supportive comments.

My major concern for this study is whether the authors have underestimated the contribution of developmentally regulated changes to the cellular and network responses observed post sensory deprivation. The authors claim in the Introduction (line 99-101) that they have disentangled "developmentally regulated plasticity from activity-dependent plasticity in adulthood" by only including studies that use animals older than P21. First, P21 is hardly considered as entering rodent adulthood, in fact, explosive plasticity mechanisms occur during P21-45 across many brain regions. Second, this statement may be true for the olfactory system (critical period around the first postnatal week), but certainly not for the auditory and visual systems. The auditory system exhibits several sensitive developmental windows as late as P40, and the visual system critical period does not start until P21. Ample evidence in literature suggests that plasticity mechanisms are highly regulated by development during this time. Could the author explain why this factor has not been included in the meta-analysis?

We agree with the reviewer, and we apologize for the lack of clarity on this essential point.

First, we decided not to include studies in which deprivation was induced in pre-weaning animals to make the work manageable and focussed. The literature on developmental plasticity is vast and extremely diverse and while we believe that developmental plasticity is critical, we feel that its investigation warrants a dedicated study.

Second, as the reviewer indicates, we also appreciate that plasticity is heightened in juvenile animals, and happening at different rates across the three sensory systems. By weaning age an animal can live independently from its mother, but it is by no means fully mature. In fact, independently of critical period windows which change for different modalities and species, one could argue that until it reaches puberty and sexual maturity it is not. However, the systematic recording of sexual maturity is notably lacking in most studies, and worse many lack the exact age of the animal and just indicate "adult". This is in fact why we originally avoided graphing these data and why we chose weaning as cutoff since it was the only milestone that we could clearly identify across all papers. Besides providing a graph, we now acknowledge more explicitly the limitations of this choice.

Changes made:

1. We have rephrased the introduction to better capture the fact that we included studies done in juvenile animals and not only adults, pages 3-4

2. An explanation of why weaning was chosen as a cutoff age is now included in the methods on page 4 (inclusion criteria)

3. A quantification of the number of studies using post-weaning rodents younger than P40 (= juvenile) is now included on page 7 and in Figure 4B. They are a minority, and they are not skewed towards a specific sense.

4. In the result sections on common mechanisms of plasticity (pages 13-17), we have highlighted the studies performed in juvenile animals. When the methodologies and reporting variability allowed, we have drawn direct comparisons with data from fully-adult animals.

5. We have extended the discussion to overtly state the limitations of our study due to age selection, and to advocate the need of future studies fully focussed on the developmental aspects of deprivation-induced plasticity (pre-weaning vs juvenile vs fully mature) specifically in pre-cortical areas - page 19.

6. We have indicated studies using juvenile animals by starring the reference in the summary table, which we have also edited to mention all 91 studies at least once. In this way readers can immediately differentiate the findings from adults (non-starred, the majority) vs juvenile (starred) animals.
